# DAE-CFR: detecting microRNA-disease associations using deep autoencoder and combined feature representation

**DOI:** 10.1186/s12859-024-05757-y

**Published:** 2024-03-29

**Authors:** Yanling Liu, Ruiyan Zhang, Xiaojing Dong, Hong Yang, Jing Li, Hongyan Cao, Jing Tian, Yanbo Zhang

**Affiliations:** 1 Department of Health Statistics, School of Public Health, Shanxi Medical University, Taiyuan, China; 2 Department of Mathematics, Changzhi Medical College, Changzhi, China; 3 Department of Cardiology, First Hospital of Shanxi Medical University, Taiyuan, China; 4 Shanxi Provincial Key Laboratory of Major Diseases Risk Assessment, Taiyuan, China; 5 School of Health and Service Management, Shanxi University of Chinese Medicine, Jinzhong, China

**Keywords:** MiRNA-disease associations, Integrated similarity, Logistic function transformation, Deep autoencoder, Combined feature representation

## Abstract

**Background:**

MicroRNA (miRNA) has been shown to play a key role in the occurrence and progression of diseases, making uncovering miRNA-disease associations vital for disease prevention and therapy. However, traditional laboratory methods for detecting these associations are slow, strenuous, expensive, and uncertain. Although numerous advanced algorithms have emerged, it is still a challenge to develop more effective methods to explore underlying miRNA-disease associations.

**Results:**

In the study, we designed a novel approach on the basis of deep autoencoder and combined feature representation (DAE-CFR) to predict possible miRNA-disease associations. We began by creating integrated similarity matrices of miRNAs and diseases, performing a logistic function transformation, balancing positive and negative samples with *k*-means clustering, and constructing training samples. Then, deep autoencoder was used to extract low-dimensional feature from two kinds of feature representations for miRNAs and diseases, namely, original association information-based and similarity information-based. Next, we combined the resulting features for each miRNA-disease pair and used a logistic regression (LR) classifier to infer all unknown miRNA-disease interactions. Under five and tenfold cross-validation (CV) frameworks, DAE-CFR not only outperformed six popular algorithms and nine classifiers, but also demonstrated superior performance on an additional dataset. Furthermore, case studies on three diseases (myocardial infarction, hypertension and stroke) confirmed the validity of DAE-CFR in practice.

**Conclusions:**

DAE-CFR achieved outstanding performance in predicting miRNA-disease associations and can provide evidence to inform biological experiments and clinical therapy.

**Supplementary Information:**

The online version contains supplementary material available at 10.1186/s12859-024-05757-y.

## Background

MiRNAs are endogenous 22 nucleotide-long noncoding RNA strands that are widely found in plants, viruses, animals and humans [1, 2]. They manipulate gene expression by base pairing with partially complementary mRNA [3, 4]. Mounting evidence points to miRNA’s vital role in various bioprocesses, such as immune response [5], cell proliferation [6], tumor invasion [7], and metabolism [8]. Predicting novel miRNA-disease associations can aid understanding of complex disease mechanisms, which in turn can help to prevent, diagnose, and treat diseases [9, 10]. Additionally, understanding the role of miRNA on disease pathogenesis may contribute to the development of personalized medicines [11, 12], and advance medical progress overall. Given its significance, the identification of latent miRNA-disease interactions has become a prevalent area of academic research [13].

MiRNAs whose expression is associated with various diseases have been investigated using biological experimental methods, such as quantitative reverse transcription, microarray analysis, and deep sequencing [14]. However, biological experiments are slow, laborious, and costly, with uncertain outcomes. Using a large amount of laboratory-generated data, researchers have constructed many computing models to infer underlying miRNA-disease interactions. Of those developed thus far, these computing models can be basically summarized into two types: network-based and machine learning-based approaches. Network-based approaches are mainly based on the biological hypothesis that functionally similar miRNAs prefer to associate with phenotypically similar diseases and vice versa [15]. Chen et al. [16] presented a model named RWRMDA that used restart random walk to forecast miRNA-disease interactions. The authors applied global network similarity measurements for the first time and implemented a random walk on functional similarity network of miRNA. Gu et al. [17] designed a network consistent projection method (NCPMDA) to infer possible miRNA-disease pairs using miRNA-disease association network, miRNA similarity network and disease similarity network. Qu et al. [11] developed the KATZMDA model in which the KATZ algorithm was applied to a heterogeneous network composed of the association network and integrated similarity networks. Dai et al. [18] proposed LWBRW to infer the potential miRNA-disease interactions, a model that operated a logistic function transformation on the similarity networks and then applied bi-random walks on the miRNA and disease network. Ha [19] introduced SMAP, an efficient computational strategy for identifying miRNA-disease pairs. This approach utilized existing miRNA-disease associations to construct the matrix factorization model, incorporating comprehensive similarity measures for both miRNAs and diseases.

Network-based algorithms can mine the unknown miRNA-disease relationships by extracting topological information from association and similarity networks. While they have been proven to be effective for analyzing small-scale data, their computational complexity increases substantially as the network scale expands [20]. Therefore, it can be challenging to apply network algorithms to large-scale data, such as those involving miRNA-disease associations, which can comprise a considerable number of nodes and edges. Additionally, it is difficult to acquire an accurate prediction of the overall associations landscape because known associations are sparse in the network and limit the spread of information [21].

Machine learning-based algorithms usually use known miRNA-disease associations as positive samples, randomly select some unknown associations as negative samples, and then predict the unknown associations using training classifiers. For example, Chen et al. [22] developed the RFMDA algorithm to infer miRNA-disease interactions, which reduced dimension of sample space using a filter-based approach, and finally employed the random forest (RF) classifier for training. In another model, Zhao et al. [23] used *k*-means clustering to solve sample imbalance problem in data processing and then proposed the ABMDA model based on the Adaptive Boosting (AdaBoost) algorithm to forecast miRNA-disease interactions, which enhanced the classification accuracy. Zhou et al. [24] proposed GBDT-LR, which balanced the positive and negative samples by using *k*-means clustering, then extracted the novel features using the Gradient Boosting Decision Tree (GBDT) method, and finally used the Logistic Regression (LR) classifier to infer the scores of each miRNA-disease pair. In recent years, deep learning technology has developed rapidly in the field of bioinformatics. Liu et al. [25] presented the DFELMDA method. The authors proposed a novel feature representation strategy and then employed deep autoencoder for low-dimensional feature extraction for each miRNA and disease. Finally, the model used RF classifier to predict novel miRNA-disease pairs. Chen et al. [26] developed the DBNMDA model using deep-belief network (DBN) to infer miRNA-disease associations, which contained two parts: pre-training restricted Boltzmann machines, and fine-tuning DBN. Ha et al. [27] presented a novel approach called NCMD for predicting miRNA-disease associations. This method utilized node2vec to create low-dimensional vector representations of miRNAs and diseases. It then incorporated a deep learning framework that combined the linearity of generalized matrix factorization with the nonlinearity of a multilayer perceptron (MLP). Although these machine learning algorithms performed well, there are still several drawbacks. For instance, in previous studies, selecting negative samples was a problem; acquiring the appropriate feature representation of each miRNA-disease pair for model prediction is challenging.

Building on previous studies, we introduce more effective biological information, consider the problem of sample imbalance, and adopt a suitable feature representation strategy to enhance the model prediction ability. For the manuscript, we built a deep learning framework using Deep AutoEncoder and Combined Feature Representation (DAE-CFR) to identify hidden miRNA-disease associations. First, according to the known miRNA-disease associations, we computed the integrated similarity of miRNAs and diseases by employing the Gaussian interaction profile (GIP) kernel similarity and functional similarity for miRNAs, and GIP kernel similarity along with two kinds of semantic similarity for diseases. After applying the logistic function transformation to the two integrated similarity matrices, we addressed sample imbalance using *k*-means clustering and subsequently constructed training samples. Next, the deep autoencoder method was used to extract latent features for miRNAs and diseases, considering two types of features: the original association feature and similarity feature. Finally, we combined these latent features to form the feature representation for each miRNA-disease pair, which were then fed into the LR classifier to predict unknown associations, with model performance measured using five and tenfold cross-validation (CV). We compared DAE-CFR with six popular algorithms and nine classifiers, and the experimental results showed that DAE-CFR performed excellently. Additionally, our model was validated on another dataset to ensure its robustness. Case studies on myocardial infarction, hypertension and stroke further illustrated its effectiveness and practicability. The final pathway analysis confirmed its capability to identify disease-associated miRNAs, providing insights into their roles in diseases.

## Materials and methods

### Known human miRNA-disease associations

The known associations between miRNA and disease were downloaded from the HMDD v2.0 database [28], in which the associations have been experimentally validated. After data cleaning, 495 miRNAs, 383 diseases, and 5430 associations were obtained, as reported in the reference [25] (see Additional file 1). Let ${n}_{m}$ and ${n}_{d}$ denote the quantity of miRNAs and diseases, respectively; $A={\{{a}_{ij}\}}_{{n}_{m}\times {n}_{d}}$ represents the association matrix, where ${a}_{ij}$ is described below:

1 $${a}_{ij}=\left\{\begin{array}{ll}1, & \quad miRNA\, {m}_{i}\, is\, associated\, with\, disease\,{d}_{j}\\ 0,& \quad otherwise\end{array}\right.$$

### GIP kernel similarity of miRNAs and diseases

The GIP kernel similarity is a widely used metric in the biomedical field [29]. The association matrix has been used to calculate the GIP kernel similarity [30]. We calculated the GIP kernel similarity between miRNAs ${m}_{i}$ and ${m}_{j}$ using the following formulae:

2 $$KM\left( {m_{i} ,m_{j} } \right) = {\text{exp}}\left( { - \gamma_{m} \parallel IP\left( {m_{i} } \right) - IP\left( {m_{j} } \right)\parallel^{2} } \right)$$

3 $$\gamma_{m} = {{\gamma_{m}^{\prime } } \mathord{\left/ {\vphantom {{\gamma_{m}^{\prime } } {\left( {\frac{1}{{n_{m} }}\mathop \sum \limits_{i = 1}^{{n_{m} }} \parallel IP\left( {m_{i} } \right)\parallel^{2} } \right)}}} \right. \kern-0pt} {\left( {\frac{1}{{n_{m} }}\mathop \sum \limits_{i = 1}^{{n_{m} }} \parallel IP\left( {m_{i} } \right)\parallel^{2} } \right)}}$$

where ${\gamma }_{m}$ controls kernel bandwidth and $IP({m}_{i})$ denotes the *i*-th row of $A$. Similarly, for disease ${d}_{i}$ and disease ${d}_{j}$, the GIP kernel similarity is computed as below:

4 $$KD\left( {d_{i} ,d_{j} } \right) = {\text{exp}}\left( { - \gamma_{d} \parallel IP\left( {d_{i} } \right) - IP\left( {d_{j} } \right)\parallel^{2} } \right)$$

5 $$\gamma_{d} = {{\gamma_{d}^{\prime } } \mathord{\left/ {\vphantom {{\gamma_{d}^{\prime } } {\gamma_{d}^{\prime } \left( {\frac{1}{{n_{d} }}\sum\limits_{i = 1}^{{n_{d} }} \parallel IP\left( {d_{i} } \right)\parallel^{2} } \right) \, }}} \right. \kern-0pt} { \left( {\frac{1}{{n_{d} }}\sum\limits_{i = 1}^{{n_{d} }} \parallel IP\left( {d_{i} } \right)\parallel^{2} } \right) \, }}$$

where ${\gamma }_{d}$ controls kernel bandwidth and $IP({d}_{i})$ represents the *i*-th row of ${A}^{T}$. We set ${\gamma }_{m}^{\mathrm{^{\prime}}}=1$ and ${\gamma }_{d}^{\mathrm{^{\prime}}}=1$ according to the references [31, 32].

### Functional similarity of miRNAs

According to the hypothesis that functionally similar miRNAs prefer associating with similar diseases, Wang et al. [33] calculated the functional similarity between miRNAs. These data can be downloaded from http://www.cuilab.cn/files/images/cuilab/misim.zip. Let $FM$ denotes miRNA functional similarity matrix, in which the element $FM({m}_{i} ,{m}_{j})$ means the similarity value between miRNAs ${m}_{i}$ and ${m}_{j}$.

### Semantic similarity of diseases

We downloaded the relations of diseases from the Medical Subject Headings (MeSH) database (http://www.ncbi.nlm.nih.gov/) [33, 34]. Then, we constructed hierarchical directed acyclic graphs (DAGs), which are commonly applied to compute the disease semantic similarity. For a given disease *d*, $DAG\left(d\right)=(d,N(d),E(d))$, where $N(d)$ denotes the node-set containing *d*, and $E(d)$ represents the edge-set about *d*. Using two different methods from a previous study [35], we obtained two models of disease semantic similarity.

For disease *t* in DAG(*d*), its semantic contribution value to *d* is defined as

6 $$D1_{d} \left( t \right) = \left\{ {\begin{array}{*{20}ll} 1, & \quad if \,\, t = d \\ max\left\{ {\Delta *D1_{d} \left( {t^{\prime } } \right)|\, t^{\prime } \in children\; of\; t} \right\}, & \quad if\,\, t \ne d \\ \end{array} } \right.$$

where $\Delta$ is the semantic contribution factor and is often set to 0.5 [33]. After traversing all nodes in $N(d)$, the calculation of semantic value of *d* is shown below:

7 $$DV1(d)=\sum_{t\in N(d)}D{1}_{d}(t)$$

For any two diseases ${d}_{i}$ and ${d}_{j}$, the more shared nodes in their DAGs, the more similar they are. Then the semantic similarity between ${d}_{i}$ and ${d}_{j}$ is computed as follows:

8 $$SD1({d}_{i},{d}_{j})=\frac{\sum_{t\in N({d}_{i})\cap N({d}_{j})}(D{1}_{{d}_{i}}(t)+D{1}_{{d}_{j}}(t))}{DV1({d}_{i})+DV1({d}_{j})}$$

However, for a given disease *d*, the contribution of the diseases in the same layer of the DAG(*d*) is different. If the disease *t* in the DAG(*d*) appears less in other DAGs, its contribution to *d* is higher. Therefore, we adapted the model using another semantic similarity method [35] to represent *t*’s semantic contribution to* d*:

9 $$D{2}_{d}\left(t\right)=-\mathit{log}\frac{\text{ the number of DAGs including t}}{\text{the number of diseases}}$$

Similar to formulae (7) and (8), we obtained the following formulae:

10 $$DV2(d)=\sum_{t\in N(d)}D{2}_{d}(t)$$

11 $$SD2({d}_{i},{d}_{j})=\frac{\sum_{t\in N({d}_{i})\cap N({d}_{j})}(D{2}_{{d}_{i}}(t)+D{2}_{{d}_{j}}(t))}{DV2({d}_{i})+DV2({d}_{j})}$$

Finally, to better describe the disease semantic similarity, the mean value of *SD*1 and *SD*2 was calculated as follows:

12 $$SS({d}_{i},{d}_{j})=\frac{SD1({d}_{i},{d}_{j})+SD2({d}_{i},{d}_{j})}{2}$$

### Integrated similarity of miRNAs and diseases

Using the similarity matrices mentioned above, we built the integrated similarity matrices of miRNAs and diseases, which denoted by SM and SD. SM is computed based on KM and FM. SD is computed using KD and SS. Therefore, the formulae for SM and SD are as follows:

13 $$SM({m}_{i} ,{m}_{j})=\left\{\begin{array}{ll}\frac{KM\left({m}_{i} ,{m}_{j}\right)+FM\left({m}_{i} ,{m}_{j}\right)}{2},& \quad if \,\, FM({m}_{i} ,{m}_{j})\ne 0\\ KM({m}_{i} ,{m}_{j}), & \quad otherwise\end{array}\right.$$

14 $$SD({d}_{i},{d}_{j})=\left\{\begin{array}{ll}\frac{KD({d}_{i},{d}_{j})+SS({d}_{i},{d}_{j})}{2}, & \quad if\,\, SS({d}_{i},{d}_{j})\ne 0\\ KD({d}_{i},{d}_{j}), & \quad otherwise\end{array}\right.$$

### Transformation of logistic function

Logistic function transformation has been performed successfully to adjust the similarity [18, 36, 37]. The logistic function can make the small value weaker and the large value stronger, thereby providing more differentiated similarity information for subsequent predictions. The final integrated similarity of miRNAs and diseases are defined as below:

15 $$LSM({m}_{i} ,{m}_{j})=\frac{1}{1+{e}^{(c\cdot SM({m}_{i} ,{m}_{j})+d)}}$$

16 $$LSD({d}_{i},{d}_{j})=\frac{1}{1+{e}^{(c\cdot SD({d}_{i},{d}_{j})+d)}}$$

where *c* and *d* are the control parameters. In this study, we set $c\in [-15,-1]$, tuned with five and tenfold CV. *d* was set to *log*(9999) according to previous studies [18, 36, 37].

### DAE-CFR for identifying unknown miRNA-disease interactions

**Fig. 1:**
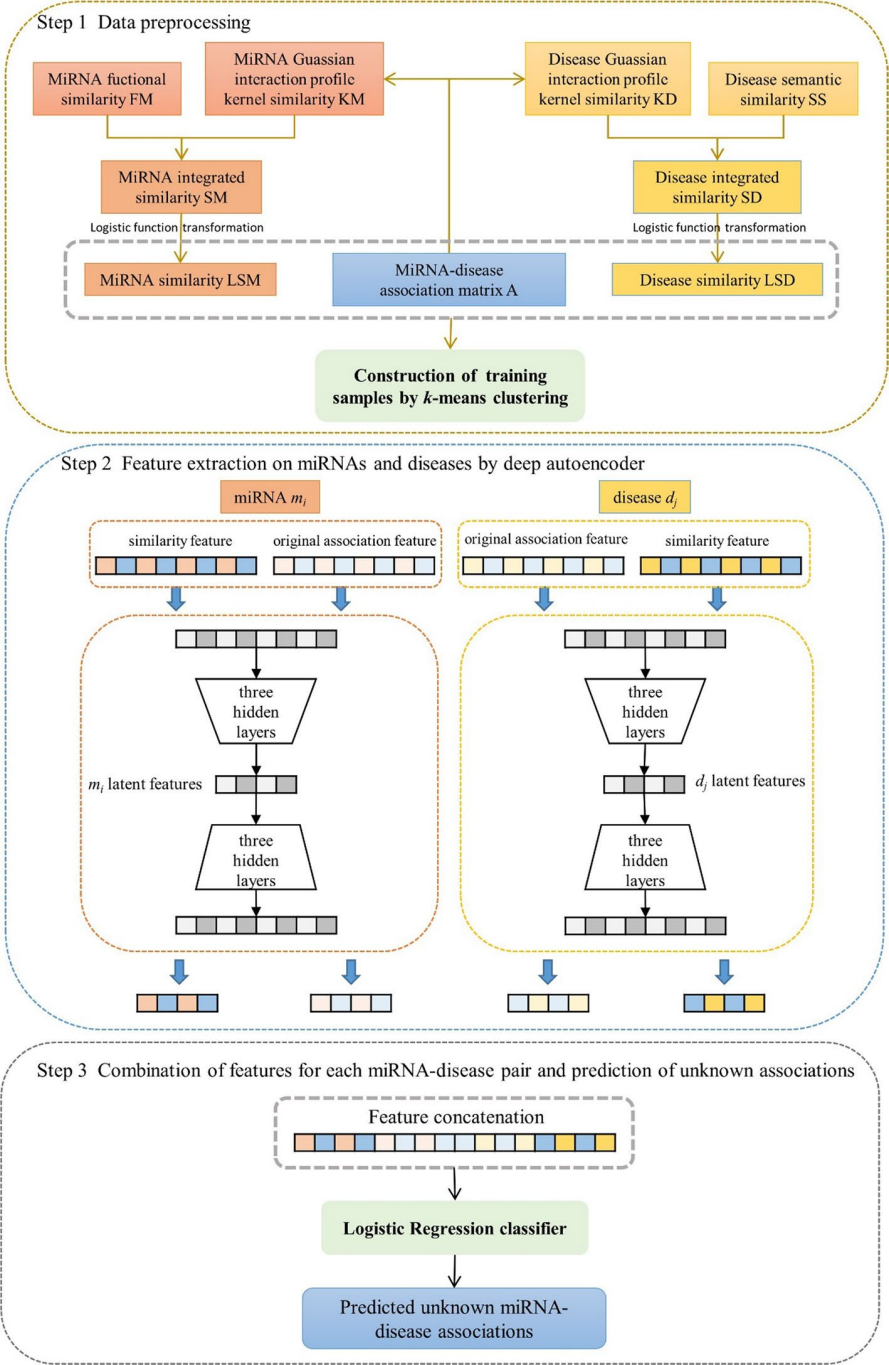
Flowchart of DAE-CFR

To identify hidden miRNA-disease interactions, we developed a novel approach using deep autoencoder and combined feature representation (DAE-CFR). The entire computation process of DAE-CFR consisted of three steps (see Fig. 1).

*Step 1* Data preprocessing.

We not only calculated similarity matrices LSM and LSD in the data preparation phase, but also constructed training samples. In this study, there were 189,585 miRNA-disease pairs consisting of 5430 known associations and 184,155 unknown associations. Here, known associations were defined as positive samples, whereas unknown associations were defined as negative samples. Since 184,155 $\gg$ 5430, there is a sample imbalance problem. To solve this problem, we introduced *k*-means clustering negative sampling, with *k* = 23 according to previous study [38]. First, we divided the negative samples into 23 groups, without making changes to the positive samples. Then we randomly chose 240 negative samples from each group so that the total number of all chosen negative samples was 5520, approximately equivalent to 5430. Overall, we obtained 10,950 training samples consisting of 5520 negative and 5430 positive samples.

*Step 2* Feature extraction on miRNAs and diseases by deep autoencoder.

Based on the original association matrix A and the similarity matrices LSM and LSD, we obtained the feature representation. In this study, we considered two types of features for each miRNA and disease (see Table 1). The first type is the original association feature: $IP({m}_{i})$ represents the association information of miRNA ${m}_{i}$ related to all diseases and $IP({d}_{j})$ denotes the association information of disease ${d}_{j}$ related to all miRNAs. The second type is similarity feature: $LSM({m}_{i})$ represents the similar information of miRNA ${m}_{i}$ with all miRNAs and $LSD({d}_{j})$ denotes the similar information of disease ${d}_{j}$ with all diseases.

**Table 1:** Feature representation of each miRNA and disease

	The type of features	Feature representation	Notation	Dimension
miRNA ${m}_{i}$	Original association feature	The *i*-th row of A	$IP({m}_{i})$	383
	Similarity feature	The *i*-th row of LSM	$LSM({m}_{i})$	495
disease ${d}_{j}$	Original association feature	The *j*-th column of A	$IP({d}_{j})$	495
	Similarity feature	The *j*-th column of LSD	$LSD({d}_{j})$	383

To represent the features more appropriately and reduce the computational complexity for subsequent prediction, we applied autoencoder to extract the low-dimensional feature representation of miRNAs and diseases. Autoencoder is an unsupervised model to recognize implicit biological patterns [39]. The autoencoder includes two phases: the encoder and the decoder [25]. In the encoding phase, the original data with high-dimensional features are compressed to low-dimensional features. In the decoding phase, the original inputs are reconstructed by mapping from the hidden layer to the output layer. The goal is to reduce the difference between the reconstructed and original data to a minimum. The autoencoder has a symmetric structure; that is, in the encoding phase, there are the same hidden layers as in the decoding phase. In this work, we used a deep autoencoder with three hidden layers to extract the latent and nonlinear features for each miRNA and disease. The deep autoencoder was implemented in the TensorFlow framework. The reduced dimensionality *L* of the latent features was set to 8, 16, 32, and 64, and we chose the proper dimensionality by comparing the effects of different *L* values. We set the batch size to 100 and used the Adam optimizer.

*Step 3* Combination of features for each miRNA-disease pair and prediction of unknown associations.

The low-dimensional and latent features of miRNAs and diseases were extracted by deep autoencoder in the last step, which we denoted as ${IP}_{1}\left({m}_{i}\right)$, ${LSM}_{1}\left({m}_{i}\right)$, ${IP}_{1}\left({d}_{j}\right)$ and ${LSD}_{1}({d}_{j})$ corresponding to the four features in Table 1, respectively. Then, we concatenated the four parts and obtained the feature representation for each miRNA-disease association as follows:

17 $$Vec\left({m}_{i},{d}_{j}\right)=[{IP}_{1}\left({m}_{i}\right),{LSM}_{1}\left({m}_{i}\right),{IP}_{1}\left({d}_{j}\right),{LSD}_{1}({d}_{j})]$$

The dimension of $Vec\left({m}_{i},{d}_{j}\right)$ is 4*L*, as each part is *L*-dimensional.

Finally, the above constructed features for each miRNA-disease pair were entered into the LR classifier to infer possible associations. These associations were then ranked by their predicted scores, with higher scores giving a higher rank. Pairs with higher scores are considered more likely to exist.

## Results

### Performance evaluation

*K*-fold CV has been widely used to assess model performance. In *k*-fold CV, the dataset is divided into *k* equal parts at random, with one part for testing and the residual parts for training. Each part takes turns as a test set, and once all *k* parts have served as the test set, the average result of all *k* test sets is used as the final evaluation. Here, we used five and tenfold CV. AUC was used as the model evaluation index and AUC $\in [\mathrm{0,1}]$. A larger value of AUC indicates better model performance. Beyond AUC, we adopted several well-established metrics. These include the F1 score, which balances precision and recall; Accuracy (ACC), indicating the proportion of correctly predicted observations to the total observations; Area Under the Precision-Recall Curve (AUPR), reflecting both the precision and recall of the model; and the Matthews Correlation Coefficient (MCC), for comprehensive class performance assessment. Together, these metrics offer a multifaceted evaluation of the model’s predictive performance and effectiveness.

### Parameters analysis

There are two parameters in the overall model: $c\in {\mathbb{Z}}^{-}$ in the logistic function transformation and $L\in {\mathbb{Z}}^{+}$ in the deep autoencoder. In this work, we considered the following value ranges: $c\in [-15,-1]$ [18] and $L\in \{\mathrm{8,16,32,64}\}$, and repeated the experiment 10 times. The grid search algorithm was adopted to choose the best parameter values. After performing the calculations and comparing the results, we obtained the optimal parameters for fivefold CV framework: *c* = − 8 and *L* = 8 (see Additional file 3: Table S1). In tenfold CV (see Additional file 3: Table S2), the best parameters were found to be *c* = − 8 and *L* = 16. When *c* = − 8 and *L* = 8, the AUC value ranked second. Therefore, for the convenience, we set *c* = − 8 and *L* = 8 both in five and tenfold CV.

### Comparison with other algorithms

To illustrate the excellent performance, DAE-CFR was compared with six popular algorithms: ABMDA [23], GBDT-LR [24], DFELMDA [25], KATZMDA [11] NCPMDA [17], and LWBRW [18]. We chose specific parameter settings for each model as described in the original study and all model parameters were listed in Additional file 3: Table S3.

**Fig. 2:**
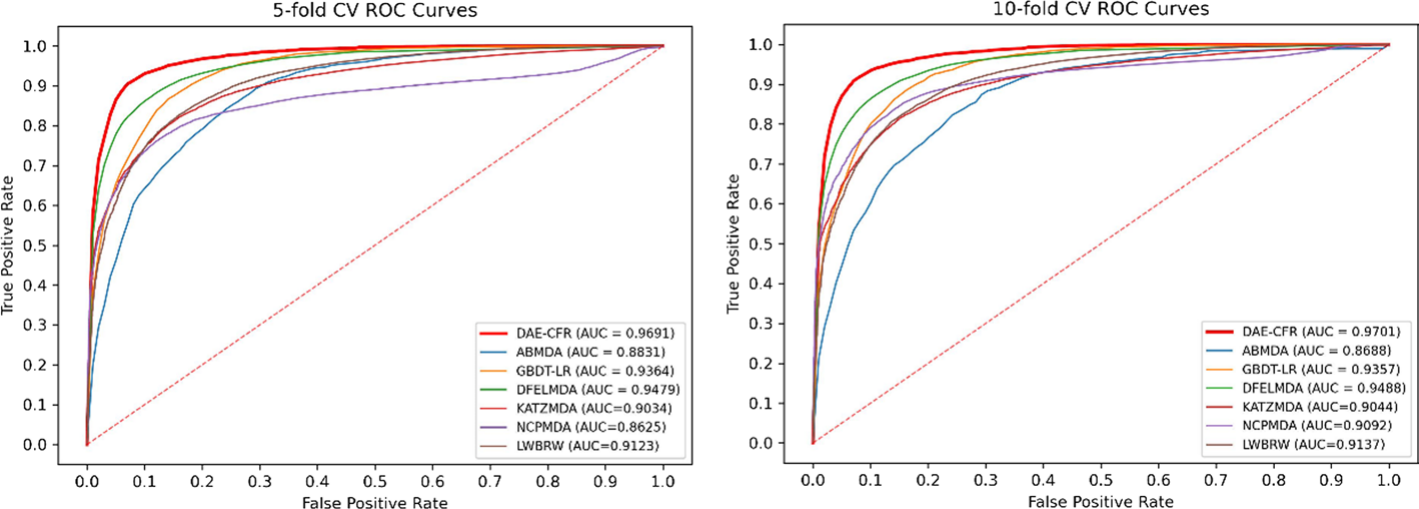
ROC curves and AUCs of seven algorithms

We conducted fivefold CV on the dataset and the AUC values are shown in Fig. 2. The AUC of DAE-CFR reached 0.9691, which exceeded the AUCs of other algorithms (ABMDA: 0.8831, GBDT-LR: 0.9364, DFELMDA: 0.9479, KATZMDA: 0.9034, NCPMDA: 0.8625, LWBRW: 0.9123). The results of all methods across various metrics were presented in Additional file 3: Table S4. The table highlighted the best-performing values for each metric in bold. From this comparison, it was evident that DAE-CFR outperformed all other methods evaluated. We then performed tenfold CV and obtained the AUC values of 0.9701, 0.8688, 0.9357, 0.9488, 0.9044, 0.9092, and 0.9137 for DAE-CFR, ABMDA, GBDT-LR, DFELMDA, KATZMDA, NCPMDA, and LWBRW, respectively. Therefore, our proposed DAE-CFR method exhibited excellent performance both in five and tenfold CV.

In addition, to ensure the robustness of the results, we repeated the experiment 10 times. The mean and standard deviation of AUCs for each method in five and tenfold CV were calculated and shown in Table 2. Here, DAE-CFR exhibited stable and superior performance compared to the six other methods, evidenced by low standard deviations and consistently higher AUC scores. This combination of reliability and effectiveness highlights its robustness in accurately predicting miRNA-disease associations, illustrating its value in biomedical research.

**Table 2:** Average AUCs of seven algorithms with 10 repeats

	AUC (fivefold CV)	AUC (tenfold CV)
DAE-CFR	0.9692 ± 0.0014	0.9696 ± 0.0012
ABMDA	0.8776 ± 0.0080	0.8639 ± 0.0080
GBDT-LR	0.9358 ± 0.0018	0.9362 ± 0.0017
KATZMDA	0.9032 ± 0.0005	0.9042 ± 0.0002
DFELMDA	0.9478 ± 0.0013	0.9484 ± 0.0020
NCPMDA	0.8638 ± 0.0014	0.9091 ± 0.0003
LWBRW	0.9130 ± 0.0004	0.9140 ± 0.0002

### Comparison with other classifiers

**Fig. 3:**
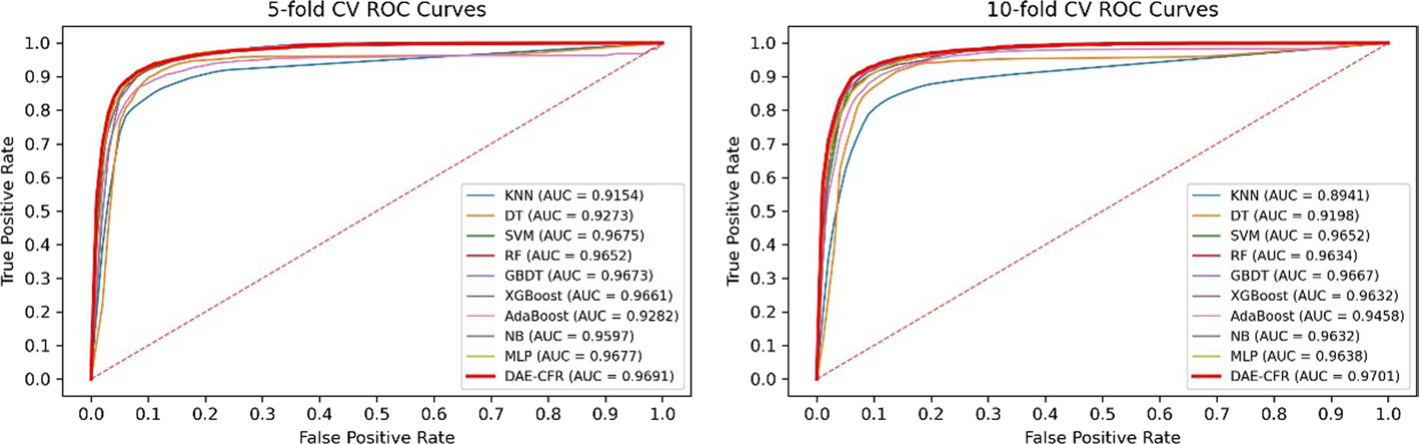
The ROC curves of different classifiers

In our model, we used LR classifier in the final step. To test the effectiveness of this choice, we replaced LR with the following nine common supervised learning classifiers: K-Nearest Neighbor (KNN), Decision Tree (DT), Support Vector Machine (SVM), RF, GBDT, eXtreme Gradient Boosting (XGBoost), AdaBoost, Naive Bayesian (NB) and MLP. Through calculations and subsequent comparisons, we derived the AUCs for various classifiers, as shown in Fig. 3. The computational results indicated that DAE-CFR outperformed the other nine classifier models, demonstrating that LR is particularly well-suited for DAE-CFR. We also repeated the experiment 10 times (see Table 3), and the results showed the stability of each method, further emphasizing the superiority of our method.

**Table 3:** Comparison of ten classifiers with 10 repeats

	AUC (fivefold CV)	AUC (tenfold CV)
LR (DAE-CFR)	**0.9692 ± 0.0014**	**0.9696 ± 0.0012**
KNN	0.9036 ± 0.0074	0.9082 ± 0.0050
DT	0.9243 ± 0.0041	0.9216 ± 0.0037
SVM	0.9649 ± 0.0014	0.9658 ± 0.0010
RF	0.9651 ± 0.0017	0.9617 ± 0.0018
GBDT	0.9644 ± 0.0022	0.9639 ± 0.0021
XGBoost	0.9642 ± 0.0015	0.9606 ± 0.0017
AdaBoost	0.9199 ± 0.0236	0.9148 ± 0.0197
NB	0.9619 ± 0.0011	0.9619 ± 0.0011
MLP	0.9435 ± 0.0231	0.9542 ± 0.0097

### Ablation study

**Fig. 4:**
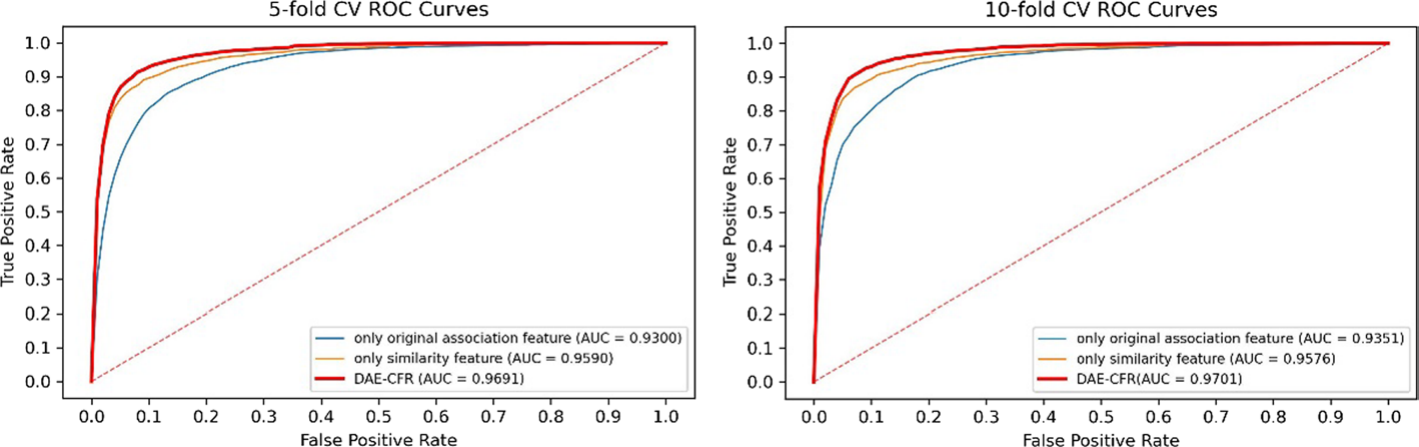
The ROC curves in the ablation study

In the work, we constructed the feature representation of each miRNA and disease using two types of features: the original association feature and the similarity feature. We compared DAE-CFR with the following two models: (1) a model with only the original association feature; (2) a model with only the similarity feature. The combined information achieved the best performance in inferring the underlying miRNA-disease pairs, as depicted in Fig. 4. Furthermore, the results confirmed the stability with 10 repeats (see Table 4).

**Table 4:** The result of the ablation study with 10 repeats

	AUC (fivefold CV)	AUC (tenfold CV)
Only original association feature	0.9293 ± 0.0050	0.9330 ± 0.0039
Only similarity feature	0.9586 ± 0.0009	0.9583 ± 0.0011
DAE-CFR	**0.9692 ± 0.0014**	**0.9696 ± 0.0012**

**Fig. 5:**
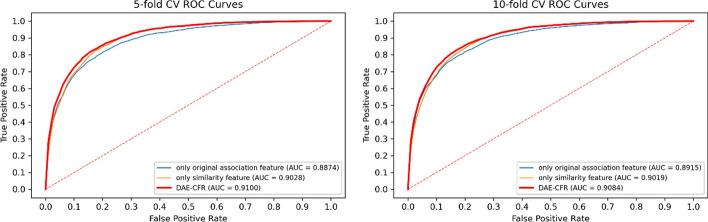
The ROC curves without the logistic function transformation in the ablation study

In the paper, we conducted the logistic function transformation in the similarity calculation, greatly enhancing model’s performance. To illustrate this, we performed experiments without the logistic function transformation, as presented in Fig. 5, where DAE-CFR still performed best. The results with logistic function transformation in Fig. 4 surpassed those in Fig. 5, demonstrating the critical importance of the logistic function transformation in model building for better performance.

### Robustness of DAE-CFR on another dataset

**Fig. 6:**
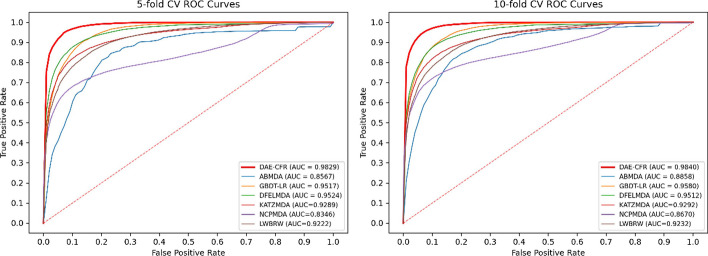
The ROC curves of seven models using the HMDD v3.2 database

To test its ability to maintain outstanding performance, we applied the DAE-CFR model to an additional dataset. For this validation, we utilized the HMDD v3.2 database [40] to extract known interactions between miRNAs and diseases. Following the data refinement, a total of 8,968 known interactions involving 374 diseases and 788 miRNAs were selected, as detailed in the reference [41] (see Additional file 2). Implementing the same experimental setup as before, the results shown in Fig. 6 indicated that DAE-CFR achieved an AUC of 0.9829 in fivefold CV, surpassing the AUCs of ABMDA (0.8567), GBDT-LR (0.9517), DFELMDA (0.9524), KATZMDA (0.9289), NCPMDA (0.8346), and LWBRW (0.9222). In tenfold CV, DAE-CFR reached an AUC score of 0.9840, outperforming the AUCs of ABMDA (0.8858), GBDT-LR (0.9580), DFELMDA (0.9512), KATZMDA (0.9292), NCPMDA (0.8670), and LWBRW (0.9232). The superior performance achieved on the HMDD v3.2 database highlights the consistency and robustness of our model.

### Case studies

To further verify the accuracy and validity of DAE-CFR, we conducted case studies on myocardial infarction (MI), hypertension (HTN) and stroke. In the study, after calculating the predicted scores, we ranked all unknown pairs and listed the top 10 miRNAs for each of the three diseases in Table 5. Subsequently, the predicted miRNAs were verified using the RNADisease database [42].

**Table 5:** Top 10 miRNAs related to three diseases predicted by DAE-CFR

Diseases	Rank	MiRNA	Predicted score	Evidence
Myocardial Infarction	1	hsa-mir-146a	0.9895	RNADisease
	2	hsa-mir-221	0.9867	RNADisease
	3	hsa-mir-125b	0.9855	RNADisease
	4	hsa-mir-145	0.9839	RNADisease
	5	hsa-mir-17	0.9784	RNADisease
	6	hsa-mir-16	0.9770	RNADisease
	7	hsa-mir-222	0.9721	RNADisease
	8	hsa-mir-200c	0.9711	RNADisease
	9	hsa-let-7a	0.9709	Unknown
	10	hsa-mir-181a	0.9664	RNADisease
Hypertension	1	hsa-mir-146a	0.9862	RNADisease
	2	hsa-mir-34a	0.9821	RNADisease
	3	hsa-mir-221	0.9815	RNADisease
	4	hsa-mir-125b	0.9801	Unknown
	5	hsa-mir-126	0.9752	RNADisease
	6	hsa-mir-16	0.9686	RNADisease
	7	hsa-mir-222	0.9623	RNADisease
	8	hsa-mir-200c	0.9609	Unknown
	9	hsa-let-7a	0.9607	Unknown
	10	hsa-mir-143	0.9603	RNADisease
Stroke	1	hsa-mir-21	0.9995	RNADisease
	2	hsa-mir-146a	0.9824	RNADisease
	3	hsa-mir-34a	0.9771	RNADisease
	4	hsa-mir-221	0.9759	RNADisease
	5	hsa-mir-125b	0.9744	RNADisease
	6	hsa-mir-126	0.9684	RNADisease
	7	hsa-mir-20a	0.9640	RNADisease
	8	hsa-mir-17	0.9624	RNADisease
	9	hsa-mir-16	0.9598	RNADisease
	10	hsa-mir-29a	0.9545	RNADisease

MI is a significant component of the global cardiovascular disease burden, leading to increased hospital admissions and substantial financial implications all over the world [43]. MiRNAs have been found to be circulating biomarkers for the diagnosis and prevention of MI [43–46]. The top 10 predicted MI-related miRNAs were shown in Table 5, with 9 out of 10 verified by the RNADisease database. The “unknown” miRNA hsa-let-7a maybe a MI-related biomarker. We conducted a literature search on PubMed and found two articles for hsa-let-7a related to MI. Du et al. [47] found that hsa-let-7a controls the expression of β1-AR and establishes a negative feedback mechanism within the β1-AR signaling pathway in cases of ischemic heart failure. This discovery offers a fresh perspective on the differences in β1-AR expression between the early and later stages of MI. According to Gan et al. [48], the circRNA-101237/let-7a-5p/IGF2BP3 axis, which plays a role in controlling cardiomyocyte death, presents potential as a promising therapeutic target for addressing cardiovascular diseases, including MI. These two clues imply that hsa-let-7a is a promising biomarker of MI and may be confirmed by further biological experiment.

The impact of HTN on public health and the economy is far beyond the scope of HTN treatment [49]. HTN, as a common chronic disease that affects the aging population [50], is a risk factor for many diseases including cardiovascular disease [51], chronic kidney disease [52] and so on, which severely threatens human life and health. Several miRNAs have been identified as potential HTN biomarkers [53, 54]. In the study, 7 out of 10 HTN-related miRNAs confirmed by the RNADisease database, as shown in Table 5. The “unknown” miRNAs (hsa-mir-125b, hsa-mir-200c and hsa-let-7a) may be novel biomarkers. We conducted a search on PubMed and identified one paper on hsa-let-7a related to HTN. Through an investigation into the roles of brain microvascular pericyte-derived extracellular vesicle miRNAs in HTN, Wu et al. [55] identified specific miRNAs like miR-21-5p, let-7c-5p, and let-7a-5p that showed abnormal expression in spontaneously hypertensive rats compared to normotensive rats. This study sheds light on the connection between brain microvascular pericytes and HTN. It suggests that hsa-let-7a is a more likely biomarker for HTN.

Stroke is a significant global cause of both mortality and disability, affecting people worldwide [56]. Notably, 87% of all strokes are ischemic in nature [57]. Researchers have explored the potential of miRNAs as biomarkers for diagnosing, predicting outcomes, and assessing brain injury in ischemic strokes [58–60]. Here, we employed DAE-CFR to identify miRNAs associated with stroke and selected the top 10 candidates. Consequently, all of the 10 miRNAs were confirmed by the RNADisease database (see Table 5).

### Pathway analysis

**Fig. 7:**
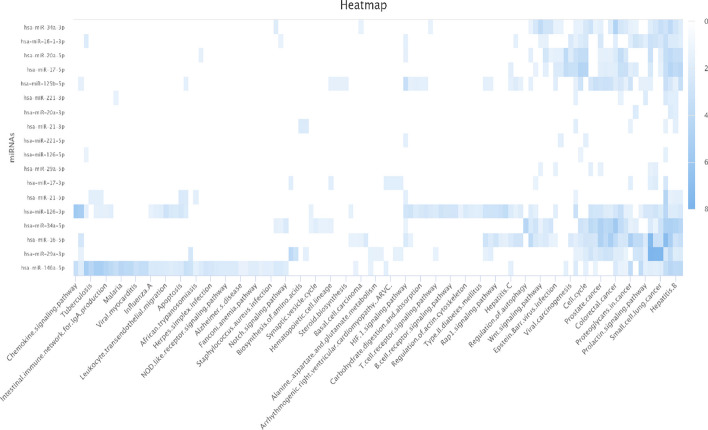
The heatmap of Stroke-related miRNAs

Inspired by references [61] and [19], we recognized the importance of in-depth pathway analysis in understanding the role of miRNAs in disease incidence. Therefore, we employed DIANA-miRPath v4.0 [62], an online platform for miRNA target and pathway analysis, to explore the regulatory functions of miRNAs and their impact on various pathways, shedding light on their connections to diseases. Specifically, we illustrated, using stroke as an example, how the majority of miRNA targets identified through the DAE-CFR method are linked to biological processes and functionalities relevant to stroke. Details of the top 10 enrichment results for stroke-associated candidate miRNAs were listed in Table 6. Research has underscored the neuroprotective role of the PI3K-Akt signaling pathway in ischemic stroke [63]. Furthermore, an association has been found between hepatitis B virus infection and a decreased risk of ischemic stroke [64]. In Fig. 7, a heatmap was presented, created using miRpathDB v2.0 [65], to show the associations between miRNA targets and their respective pathways, where a darker shade indicated a stronger association with pathway functions. This pathway analysis not only validated the reliability and efficiency of the DAE-CFR method in identifying disease-related candidate miRNAs but also provided valuable insights into the role of miRNAs in diseases.

**Table 6:** TOP 10 Enrichment results for Stroke-related candidate miRNAs

KEGG pathway	*p*-value
Pathways in cancer	1.24e-126
MicroRNAs in cancer	1.22e-86
PI3K-Akt signaling pathway	1.05e-72
Breast cancer	1.14e-70
Hepatitis B	1.22e-70
Pancreatic cancer	1.23e-70
AGE-RAGE signaling pathway in diabetic complications	1.27e-70
EGFR tyrosine kinase inhibitor resistance	1.08e-67
Proteoglycans in cancer	9.42e-67
Prostate cancer	3.76e-65

## Discussion

In this study, we developed a novel deep learning framework DAE-CFR for predicting hidden associations between miRNAs and diseases. The computational results indicated that DAE-CFR achieved outstanding performance in both five and tenfold CV. There are several reasons why DAE-CFR performed excellently. First, we introduced more biological information into the model. We computed integrated similarity of miRNAs and diseases using two sources of miRNA similarity and three types of disease similarity, respectively. Second, we applied the logistic function transformation to the two integrated similarity matrices to improve the discrimination of the similarity information. Third, we adopted *k*-means clustering negative sampling, which is simple and efficient for large-scale datasets. Fourth, the use of deep autoencoder achieved feature dimension reduction and improved computational efficiency. We considered both the original association feature and similarity feature for miRNAs and diseases and found that the combined features outperformed the singular features in the ablation study. Due to all of these factors, our model significantly enhanced forecasting performance.

Despite its numerous strengths, DAE-CFR has limitations that require further exploration and refinement. First, GIP kernel calculations are essentially based on current miRNA-disease associations. The limited number of known interactions could bias the predictive results. This scarcity of confirmed associations might lead models to overvalue the few recognized links, potentially neglecting unexplored or novel interactions. Second, we treated known associations as positive samples and considered all unknown associations as negative samples, facing the challenge of acquiring credible negative samples, inherently difficult to obtain in biological systems. This strategy may risk neglecting or misclassifying potential interactions, further illustrating the limitations of relying only on known miRNA-disease relationships. Third, in our method, we did not consider how changes in the relationships between miRNAs and diseases might affect model performance. This consideration is crucial for ensuring that our model remains effective as association data are updated. Forth, our analysis has focused solely on miRNAs as the biological determinant in disease pathogenesis, overlooking the roles of other biological entities, such as proteins and lncRNAs, which also influence disease mechanisms. Future research should not only extend this methodological framework to more miRNA-disease association datasets and more prediction challenges but also aim to include sensitivity analysis to evaluate how variations in these associations affect model performance. Additionally, we plan to broaden the investigative scope to encompass a wider array of biological entities and their interactions. This comprehensive approach will enrich our understanding and enhance the accuracy of our predictions.

## Conclusion

Previous studies have found that miRNAs are critical in disease processes. Inferring unknown miRNA-disease interactions can increase our understanding of the pathogenesis of complex human diseases, contributing to their prevention and therapy. In recent years, the identification of miRNA-disease associations has increased significantly owing to the growth of experimental technologies. However, laboratory methods can be time-consuming and laborious. Therefore, many researchers have developed algorithms to forecast potential miRNA-disease pairs. In this research, we proposed the DAE-CFR method, which employs deep autoencoder for complex feature extraction and utilizes a combined feature representation technique. First, we computed the integrated similarity of miRNAs and diseases using GIP kernel similarity, miRNA functional similarity, and two types of disease semantic similarity; applied the logistic function transformation to obtain the final integrated similarity; balanced the positive and negative samples by *k*-means clustering and then constructed training samples. Second, we used deep autoencoder to extract latent features from two types of feature representation for each miRNA and disease: the original association feature and the similarity feature. Finally, we combined these latent features to form the feature representation for each miRNA-disease pair, and then applied the LR classifier to forecast unknown pairs. To verify the superiority of DAE-CFR, we compared it with six other popular models: ABMDA, GBDT-LR, DFELMDA, KATZMDA, NCPMDA, and LWBRW in five and tenfold CV frameworks, finding that DAE-CFR showed the best results with AUCs of 0.9691 and 0.9701, respectively. In our model, we chose LR classifier in the final prediction, which was superior to the other nine common classifiers. Subsequently, the robustness was affirmed through its validation on another dataset. Furthermore, we conducted case studies on three diseases and found that the accuracy of the top 10 predicted miRNAs for MI, HTN and Stroke was 90%, 70% and 100%, respectively. The final pathway analysis validated the DAE-CFR method’s effectiveness in identifying disease-related miRNAs and offered insights into miRNAs’ roles in diseases, enhancing our model’s predictive accuracy and biological understanding. In summary, DAE-CFR presented powerful performance in identifying miRNA-disease associations, demonstrating its significant potential in the field.

### Supplementary Information

**Additional file 1**. Known miRNA-disease associations collected from HMDD v2.0 database.

**Additional file 2**. Known miRNA-disease associations collected from HMDD v3.2 database.

**Additional file 3**. Supplementary Tables. This file includes four tables: Table S1. The AUC values of 5-fold CV in parameters analysis with 10 repeats; Table S2. The AUC values of 10-fold CV in parameters analysis with 10 repeats; Table S3. Parameter settings for all methods; Table S4. The performance comparison of different methods on 5-fold CV.

## Data Availability

The datasets used in this study are included in the article/Supplementary Material.
