# Supplementary material for: DAE-CFR: detecting microRNA-disease associations using deep autoencoder and combined feature representation
Source: BMC Bioinformatics. 2024 Mar 29;25:139. doi: 10.1186/s12859-024-05757-y (PMC10981315; doi:10.1186/s12859-024-05757-y)
Supplement: Supplementary file 3 — Additional file 3. Supplementary Tables. This file includes four tables: Table S1. The AUC values of 5-fold CV in parameters analysis with 10 repeats; Table S2. The AUC values of 10-fold CV in parameters analysis with 10 repeats; Table S3. Parameter settings for all methods; Table S4. The performance comparison of different methods on 5-fold CV. [file 12859_2024_5757_MOESM3_ESM.docx]

**Table S1**. The AUC values of 5-fold CV in parameters analysis with 10 repeats

| *c* | *L*=8 | *L*=16 | *L*=32 | *L*=64 |
| --- | --- | --- | --- | --- |
| -1 | 0.9021±0.0035 | 0.9029±0.0054 | 0.9048±0.0046 | 0.9058±0.0044 |
| -2 | 0.9157±0.0046 | 0.9158±0.0056 | 0.9172±0.0041 | 0.9157±0.0070 |
| -3 | 0.9215±0.0036 | 0.9207±0.0055 | 0.9250±0.0022 | 0.9227±0.0075 |
| -4 | 0.9236±0.0033 | 0.9236±0.0057 | 0.9266±0.0037 | 0.9294±0.0030 |
| -5 | 0.9327±0.0032 | 0.9317±0.0034 | 0.9315±0.0057 | 0.9351±0.0052 |
| -6 | 0.9588±0.0017 | 0.9582±0.0020 | 0.9578±0.0022 | 0.9565±0.0026 |
| -7 | 0.9668±0.0011 | 0.9658±0.0013 | 0.9654±0.0016 | 0.9646±0.0015 |
| -8 | **0.9690±0.0011** | 0.9686±0.0014 | 0.9677±0.0021 | 0.9679±0.0013 |
| -9 | 0.9684±0.0010 | 0.9683±0.0013 | 0.9680±0.0013 | 0.9673±0.0013 |
| -10 | 0.9655±0.0014 | 0.9641±0.0020 | 0.9641±0.0018 | 0.9633±0.0018 |
| -11 | 0.9610±0.0012 | 0.9606±0.0014 | 0.9605±0.0015 | 0.9599±0.0014 |
| -12 | 0.9635±0.0012 | 0.9625±0.0014 | 0.9639±0.0009 | 0.9624±0.0013 |
| -13 | 0.9514±0.0011 | 0.9513±0.0015 | 0.9522±0.0013 | 0.9510±0.0013 |
| -14 | 0.9480±0.0012 | 0.9474±0.0012 | 0.9465±0.0013 | 0.9460±0.0021 |
| -15 | 0.9549±0.0011 | 0.9544±0.0015 | 0.9529±0.0022 | 0.9518±0.0014 |

**Table S2**. The AUC values of 10-fold CV in parameters analysis with 10 repeats

| *c* | *L*=8 | *L*=16 | *L*=32 | *L*=64 |
| --- | --- | --- | --- | --- |
| -1 | 0.9036±0.0034 | 0.9059±0.0042 | 0.9067±0.0038 | 0.9084±0.0046 |
| -2 | 0.9135±0.0033 | 0.9161±0.0038 | 0.9199±0.0027 | 0.9198±0.0070 |
| -3 | 0.9200±0.0031 | 0.9224±0.0037 | 0.9228±0.0047 | 0.9265±0.0030 |
| -4 | 0.9205±0.0039 | 0.9235±0.0065 | 0.9280±0.0039 | 0.9256±0.0100 |
| -5 | 0.9364±0.0024 | 0.9345±0.0042 | 0.9355±0.0061 | 0.9372±0.0061 |
| -6 | 0.9604±0.0013 | 0.9600±0.0019 | 0.9589±0.0023 | 0.9594±0.0017 |
| -7 | 0.9671±0.0010 | 0.9667±0.0012 | 0.9663±0.0013 | 0.9658±0.0013 |
| -8 | **0.9691±0.0011** | **0.9694±0.0014** | 0.9685±0.0015 | 0.9682±0.0014 |
| -9 | 0.9685±0.0015 | **0.9691±0.0009** | 0.9687±0.0012 | 0.9683±0.0021 |
| -10 | 0.9654±0.0008 | 0.9656±0.0011 | 0.9654±0.0014 | 0.9649±0.0018 |
| -11 | 0.9613±0.0014 | 0.9612±0.0012 | 0.9603±0.0024 | 0.9606±0.0016 |
| -12 | 0.9636±0.0007 | 0.9644±0.0007 | 0.9636±0.0009 | 0.9633±0.0014 |
| -13 | 0.9526±0.0017 | 0.9528±0.0012 | 0.9527±0.0019 | 0.9517±0.0017 |
| -14 | 0.9481±0.0010 | 0.9477±0.0008 | 0.9475±0.0011 | 0.9467±0.0013 |
| -15 | 0.9551±0.0007 | 0.9544±0.0018 | 0.9531±0.0024 | 0.9516±0.0013 |

**Table S3**. Parameter settings for all methods

| Method | Parameter settings |
| --- | --- |
| DAE-CFR | *c=*-8, *L=*8 |
| ABMDA | *k*=23*,* *max_depth*=9*, min_samples_leaf*=5 |
| GBDT-LR | *k*=23, *n_estimators* = 12*, max_depth*=5*, min_samples_leaf*=13 |
| DFELMDA | *encoding_dim* = 64, ratio= 0.5:0.5 |
| KATZMDA | $\beta=$0.01*, k=*2 |
| NCPMDA | - |
| LWBRW | *K*=4, $\alpha$=0.4, $\eta$=0.9, *c*=-8, $I_{r}$=1, $I_{l}$=2 |

**Table S4**. The performance comparison of different methods on 5-fold CV

| Method | AUC | AUPR | ACC | F1 | MCC |
| --- | --- | --- | --- | --- | --- |
| DAE-CFR | **0.9691** | **0.9681** | 0.9165 | **0.9162** | **0.8336** |
| ABMDA | 0.8831 | 0.8704 | 0.7975 | 0.7980 | 0.5998 |
| GBDT-LR | 0.9364 | 0.9302 | 0.8613 | 0.8623 | 0.7237 |
| DFELMDA | 0.9479 | 0.5718 | **0.9774** | 0.4229 | 0.4693 |
| KATZMDA | 0.9034 | 0.0496 | 0.5641 | 0.0363 | 0.0866 |
| NCPMDA | 0.8625 | 0.0218 | 0.5241 | 0.0268 | 0.0691 |
| LWBRW | 0.9123 | 0.1049 | 0.5052 | 0.0372 | 0.0834 |
